# Lumpy skin disease, an emerging transboundary viral disease: A review

**DOI:** 10.1002/vms3.434

**Published:** 2021-02-01

**Authors:** Fatemeh Namazi, Azizollah Khodakaram Tafti

**Affiliations:** ^1^ Department of Pathobiology School of Veterinary Medicine Shiraz University Shiraz Iran

**Keywords:** capripox, epidemiology, lumpy skin disease, transboundary disease

## Abstract

Lumpy skin disease is an emerging bovine viral disease, which is endemic in most African countries and some Middle East ones, and the elevated risk of the spread of disease into the rest of Asia and Europe should be considered. The recent rapid spread of disease in currently disease‐free countries indicates the importance of understanding the limitations and routes of distribution. The causative agent, Capripoxvirus, can also induce sheeppox and goatpox. The economic significance of these diseases is of great concern, given that they threaten international trade and could be used as economic bioterrorism agents. The distribution of capripoxviruses seems to be expanding due to limited access to effective vaccines and poverty within farming communities. This is largely due to the economic effects of the Covid‐19 pandemic and the imposition of crippling sanctions in endemic regions, as well as an increase in the legal and illegal trade of live animals and animal products, and also global climate change. The present review is designed to provide existing information on the various aspects of the disease such as its clinicopathology, transmission, epidemiology, diagnosis, prevention and control measures, and the potential role of wildlife in the further spread of disease.

## Graphical abstract

Lumpy skin disease is an emerging disease, which the recent rapid spread of disease in disease‐free countries indicates the importance of understanding of the limitations and routes of distribution. The present review is designed to provide existing information on the various aspects of the disease.

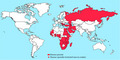

## 1 INTRODUCTION

Lumpy skin disease (LSD), a major threat to stockbreeding, can cause acute or subacute disease in cattle and water buffalo (Givens, 2018; Tuppurainen, Venter, et al., 2017). All ages and breeds of cattle are affected, but especially the young and cattle in the peak of lactation (Tuppurainen et al., 2011). The reason why the World Organization for Animal Health (OIE) has placed this transboundary disease on the notifiable disease list is due to its significant economic losses and the potential for rapid spread (Tuppurainen & Oura, 2012). The recent spread of the disease in disease‐free countries indicates the importance of its transmission, as well as control and eradication (Sprygin et al., 2019). Lumpy skin disease virus (LSDV) is a double‐stranded DNA containing around 150 kilobase pairs (kbp) with relatively large sizes (230–260 nm), enclosed in a lipid envelope and belongs to genus Capripoxvirus, which is genetically related to the sheep pox (SPPV) and goat pox (GTPV) viruses (Bhanuprakash et al., 2006; Buller et al., 2005; Givens, 2018). This virus is the most economically significant in the Poxviridae family affecting domestic ruminants. The capsid or nucleocapsid of the virus is brick or oval shaped containing the genome and lateral bodies. Extensive DNA cross‐hybridization between species causes serologic cross‐reaction and cross‐protection among members. Although Capripoxviruses are generally considered to be host specific, SPPV and GTPV strains can naturally or experimentally cross‐infect and cause disease in both host species. In contrast, LSDV can experimentally infect sheep and goats, but no natural infection of sheep and goats with LSDV has been reported.

## 2 CLINICOPATHOLOGY

**FIGURE 1:**
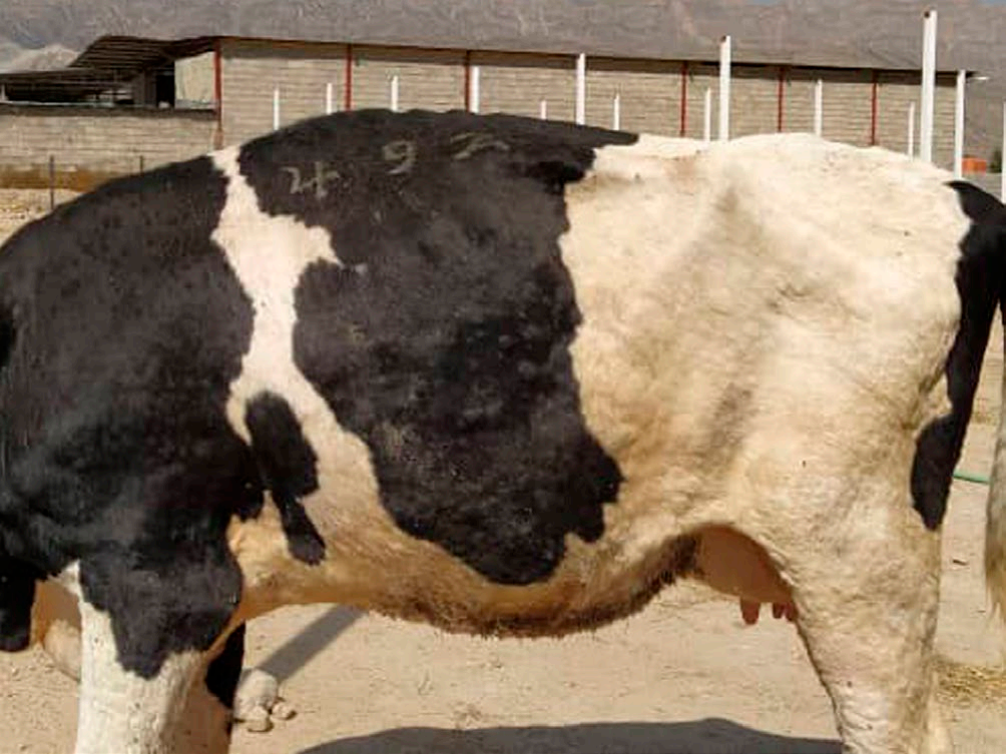
Lumpy skin disease. Raised, circumscripted nodular lesions

The clinical features of the disease include fever, inappetence, nasal discharge, salivation and lachrymation, enlarged lymph nodes, a considerable reduction in milk production, loss of body weight and sometimes death (Abutarbush et al., 2013; Annandale et al., 2014; Babiuk et al., 2008; Tasioudi et al., 2016). Furthermore, the disease is characterized by firm, slightly raised, circumscribed skin nodules (Figure 1) that are 2–7 cm in diameter and typically appear on the neck, legs, tail and back, shortly after the beginning of fever (Beard, 2016; Sevik & Dogan, 2017). The necrotic and ulcerative nodules raise the risk of myiasis (Beard, 2016). Oedema of the legs and lameness was observed in some cases (Tuppurainen & Oura, 2012). LSDV can lead to abortion (Radostitis et al., 2006), mastitis and orchitis (Awadin et al., 2011). However, nodules were not observed in aborted fetuses (Sevik & Dogan, 2017). With necropsy, lung oedema and congestion, nodules throughout the lungs and gastrointestinal tract were often observed (Zeynalova et al., 2016). Tissues such as the muzzle, nasal cavity, larynx, trachea, inside of the lips, dental pad, gingiva, abomasum, udder, teats, uterus, vagina and testes might be affected. The complications of severe disease were reported as keratitis, dysentery, lameness, pneumonia, mastitis and myiasis (Al‐Salihi & Hassan, 2015; Tuppurainen et al., 2017).

The histopathological examination of skin nodules may reveal pathognomonic eosinophilic intracytoplasmic inclusion bodies in the keratinocytes, macrophages, endothelial cells and pericytes and are associated with the ballooning degeneration of spinosum cells. Infiltration of the superficial dermal tissue of affected areas by inflammatory cells such as macrophages, lymphocytes and eosinophils is seen. In addition, widespread vasculitis and severe coagulative necrosis in subcutaneous muscles may be observed in some cases (Constable et al., 2017; Sevik et al., 2016). Pseudo‐lumpy skin disease, urticaria, streptotrichosis (*Dermatophilus congolensis* infection), ringworm, *Hypoderma bovis* infection, photosensitization, bovine papular stomatitis, foot and mouth disease, bovine viral diarrhoea and malignant catarrhal fever are all considered in the differential diagnosis of LSD (Abutarbush, 2017).

## 3 PATHOGENESIS

Following LSDV infection, virus replication, viremia, fever, cutaneous localization of the virus and development of nodules occur (Constable et al., 2017). Experimentally, after intradermal inoculation of the virus, the following events were reported:

- 4 to 7 days post‐infection (DPI): localized swelling as 1–3 cm nodules or plaques at the site of inoculation
- 6 to 18 DPI: viremia and shedding of the virus via oral and nasal discharge
- 7 to 19 DPI: regional lymphadenopathy and development of generalized skin nodules
- 42 days after fever: presence of virus in semen (Coetzer, 2004).

Intracellular replication of the virus in fibroblasts, macrophages, pericytes and endothelial cells leads to vasculitis and lymphangitis in affected tissues (Coetzer, 2004).

It seems that young calves, lactating cows and underweight animals are more susceptible to natural infections, probably due to impairment of humoral immunity (Babiuk, Bowden, Boyle, et al., 2008). Animals that have recovered from natural infection by the virus have shown lifelong immunity. Calves from their infected dams are resistant to clinical disease for approximately 6 months because of the acquired maternal antibodies (Tuppurainen et al., 2005). Affected animals clear the infection and no carrier state has known for LSDV yet (Tuppurainen, Alexandrov, et al., 2017).

## 4 TRANSMISSION

Lumpy skin disease can affect cattle, water buffalo and wild ruminants. It seems that sheep and goats are not infected by the virus (El‐Nahas et al., 2011; Lamien, Le Goff, et al., 2011). LSDV can remain viable for long periods in the environment at ambient temperatures, especially in dried scabs. It is reported that the virus persists in necrotic skin nodules for up to 33 days or longer, in desiccated crusts for up to 35 days and for at least 18 days in air‐dried hides. The virus can be inactivated at a temperature of 55°C for 2 hr and 65°C for 30 min (Mulatu & Feyisa, 2018). The main sources of infection are considered to be skin lesions as the virus persists in the lesions or scabs for long periods. The virus is also excreted via the blood, nasal and lachrymal secretions, saliva, semen and milk (transmissible to suckling calves).

The LSDV is transmitted through arthropods, particularly blood‐sucking insects (Chihota, Rennie, Kitching, & Mellor, 2001, 2003; MacLachlan & Dubovi, 2011), contaminated feed and water and direct transmission in the later stages of the disease via saliva, nasal secretions and semen (Annandale et al., 2014; Chihota et al., 2001; Irons et al., 2005; Tuppurainen, Venter, et al., 2017). Some studies showed no positive correlation between cattle density and infection rates, indicating low importance of direct virus transmission, at least in the early stages of the disease, compared with the higher significance of indirect transmission (Carn & Kitching, 1995; Magori‐Cohen et al., 2012).

As most LSD outbreaks have occurred in the summer when arthropods are most active, it may indicate the involvement of various vector species, especially blood‐feeding insects, in virus spread (Kahana‐Sutin et al., 2017; Sprygin et al., 2018).

Several studies have suggested a possible role of hard ticks in virus transmission (Lubinga et al., 2015; Tuppurainen et al., 2011, 2013). Lumpy skin disease virus and viral antigen were found in the saliva and the different organs of ticks, including the haemocytes, salivary glands and midgut in saliva and different organs of ticks such as haemocytes, salivary glands and midgut (Lubinga et al., 2013, 2014). Furthermore, the transstadial and mechanical transmission of the virus by ticks was proved based on molecular evidence (Tuppurainen & Oura, 2012). However, their prolonged attachment to the host does not explain the rapid occurrence of extensive epidemics. Therefore, it seems that ticks may be acting as reservoirs for the virus (Kahana‐Sutin et al., 2017).

*Aedes aegypti* is the sole dipteran to be able to fully transmit the virus to susceptible cattle (Chihota et al., 2001). Mosquitoes such as *Culicoides nubeculosus*, *Culex quinquefasciatus* Say and *Anopheles stephensi* Liston were not able to transmit the virus (Chihota et al., 2003).

Although *Stomoxys calcitrans* has been seen in LSD outbreaks and has transmitted the capripox virus to sheep and goats (Baldacchino et al., 2013; Yeruham et al., 1995), the transmission of LSDV to susceptible animals has failed (Chihota et al., 2003). Since LSDV has been detected in *Culicoides punctatus*, it may play a role in virus transmission (Sevik & Dogan, 2017). It is also stated that the ratio of biting insects to host population is positively correlated with transmission possibility (Gubbins et al., 2008).

In experimental studies, the persistence of lumpy skin disease virus was indicated in bovine semen by both PCR and virus isolation (Annandale et al., 2010; Givens, 2018; Irons et al., 2005). Also, semen caused the transmission of the virus to inseminated heifers (Annandale et al., 2014).

## 5 EPIDEMIOLOGY

### 5.1 Geographical distribution

**FIGURE 2:**
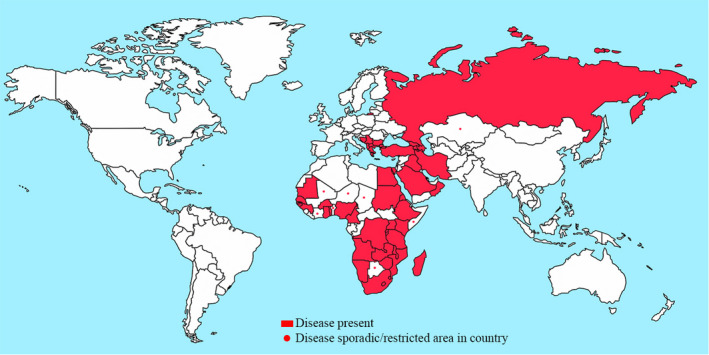
Global situation of lumpy skin disease (FAO, 2016)

LSDV was diagnosed for the first time in Zambia in 1929 and then reported in several regions of African countries (Wainwright et al., 2013). The disease has been identified in Saudi Arabia, Lebanon, Jordan, Iraq, Israel, Turkey and Iran (Abutarbush et al., 2013; Al‐Salihi & Hassan, 2015; Ben‐Gera et al., 2015; Ince et al., 2016; Sameea Yousefi et al., 2017). Since 2015, it has spread to Russia, Azerbaijan, Armenia, Greece and Bulgaria, Albania, Kosovo, Serbia and Montenegro (Beard, 2016; EFSA, 2017; OIE, 2015; Ripani & Pacholek, 2015; Tasioudi et al., 2016; Wainwright et al., 2013; Zeynalova et al., 2016). Therefore, the elevated risk of the spread of disease into the rest of Europe and Asia should be considered (Figure 2).

**FIGURE 3:**
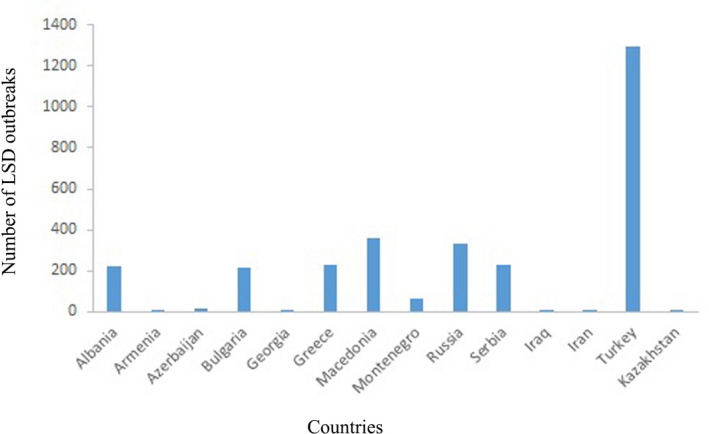
The number of LSD outbreaks in different countries during 2014–2016 (OIE, 2018)

The number of lumpy skin disease outbreaks in various countries was reported in the years 2014–2016 by the OIE (Figure 3). For instance, the numbers of LSD outbreaks in some Middle Eastern countries with extensive boundaries were 6, 8, 1,294, 1, 16, 1 and 330 in Iran, Iraq, Turkey, Kazakhstan, Azerbaijan, Armenia and Russia, respectively (OIE WAHID, 2018).

### 5.2 Morbidity and mortality

There have been no reports on the incubation period of LSDV infection under field conditions (OIE, 2018). Although the morbidity rate varies between 5% and 45% (sometimes up to 100%), the mortality rate is usually under 10% (sometimes up to 40%) (Coetzer, 2004). For instance, the morbidity and mortality rates of outbreaks were reported as 8.7% and 0.4%, respectively, in Greece (Tasioudi et al., 2016) and 12.3% and 6.4%, in Turkey (Sevik & Dogan, 2017). The severity of the clinical disease is often influenced by the animal's age, breed, immune status and production period (Tuppurainen, Venter, et al., 2017).

### 5.3 Risk factors

Risk factors associated with the spread of LSD include a warm and humid climate, conditions supporting an abundance of vector populations, such as those seen after seasonal rains, and the introduction of new animals to a herd.

The herd size, vector populations, distance to the lake, migration of herd, transport of infected animals into disease‐free areas, common pasture and water sources have all been considered as other risk factors, which may increase the disease prevalence (Gari et al., 2010; Ince et al., 2016; Sevik & Dogan, 2017). Moreover, the direction and strength of the wind may likely contribute to the virus spread (Chihota et al., 2003; Rouby & Aboulsoud, 2016).

All ages and breeds of cattle, as well as both sexes, are susceptible to the disease (Tuppurainen et al., 2011). Also, risk factors associated with LSDV seropositivity include age, sex, management type, mean annual rainfall and common water source (Ochwo et al., 2019).

### 5.4 Role of wildlife in the disease spread

Seropositivity can demonstrate the possible role of animals in the epidemiology of the disease (Barnard, 1997). It seems that mild clinical cases in wildlife are easily missed because it can be difficult or impossible to monitor the skin lesions (Barnard, 1997).

The susceptibility of springbok, impala and giraffe to the virus has been demonstrated (Lamien, Le Goff, et al., 2011; Le Goff et al., 2009; Young et al., 1970). Other species which have been seropositive for the virus include African buffaloes, blue wildebeest, eland, giraffe, impala and greater kudu (Barnard, 1997; Davies, 1982; Fagbo et al., 2014). The disease was reported in an Arabian oryx by Greth et al., (1992). However, the role of wildlife in the epidemiology of LSD is not yet well understood (Tuppurainen, Venter, et al., 2017).

## 6 ECONOMIC IMPACT

Lumpy skin disease has led to serious economic losses in affected countries. The disease causes a considerable reduction in milk yield (from 10% to 85%) due to high fever and secondary mastitis. Other consequences of the disease include damaged hides, decline of the growth rate in beef cattle, temporary or permanent infertility, abortion, treatment and vaccination costs and death of infected animals (Alemayehu et al., 2013; Babiuk, Bowden, Boyle, et al., 2008; Sajid et al., 2012; Sevik & Dogan, 2017). The total cost of the LSD outbreaks in 393 surveyed herds was 822 940.7 GBP in Turkey (Sevik & Dogan, 2017). In Ethiopia, the estimated financial loss was 6.43 USD and 58 USD per head for local zebu and Holstein Friesian, respectively (Gari et al., 2010). Total production losses resulting from the disease have been estimated at 45%–65% in industrial cattle farming (Tuppurainen & Oura, 2012). The causative agent, capripoxvirus, can induce sheeppox and goatpox as well, and these diseases have economic significance, given that they present a major hindrance to international trade and may be abused as an economic bioterrorism agent.

## 7 DIAGNOSIS

Despite a primary clinical diagnosis of LSD, the diagnosis is confirmed by using conventional PCR (Orlova et al., 2006; Tuppurainen et al., 2005; Zheng et al., 2007) or real‐time PCR techniques (Balinsky et al., 2008; Bowden et al., 2008). A real‐time PCR technique has also been established, differentiating among LSDV, sheep and goat poxviruses (Lamien, Lelenta, et al., 2011). For differentiating virulent LSDV from the vaccine strain, Restriction Fragment Length Polymorphism (RFLP) has also been used (Menasherow et al., 2014). Furthermore, electron microscopy, virus isolation, virus neutralization and serological techniques have been utilized for LSDV detection as shown in Table 1 (OIE, 2018). It is stated that molecular methods are more precise, reliable and rapid compared with other methods (Stubbs et al., 2012). Among serological techniques, the virus neutralization test, which is slow and costly with a high specificity and low sensitivity, is the only currently validated/valid test (Beard, 2016). Babiuk, Bowden, Parkyn, et al. (2008) established immunohistochemical detection of LSDV antigen in an experimental study.

**TABLE 1:** Different techniques for LSD diagnosis

Techniques	Purposes					
	Animals freedom from infection	Animal freedom from infection previous to movement	Contribution in eradication policies	Confirmation of clinical cases	Prevalence of infection surveillance	Immune status post‐vaccination
	Identification of agent					
Virus isolation	+	++	+	+++	+	‐
PCR	++	+++	++	+++	+	‐
Electron microscopy	‐	‐	‐	+	‐	‐
	Immune response detection					
Virus neutralization	++	++	++	++	++	++
Electron microscopy	+	+	+	+	+	+

Note

−: not appropriate for the purpose; +: may be used in some situations, but its application is limited by some factors such as reliability, cost, etc.; ++: appropriate method; +++: recommended method.

IFAT indicates Indirect Fluorescent Antibody Test; and PCR, polymerase chain reaction.

Despite the specificity and sensitivity of the western blot test, it is expensive and difficult to perform (OIE, 2018).

## 8 PREVENTION AND CONTROL

The distribution of capripoxviruses seems to be expanding due to limited access to effective vaccines and poverty in farming communities in endemic regions, as well as the increased legal and illegal trading of live animals, besides global climate changes. Vaccination is the only effective method to control the disease in endemic areas along with movement restrictions and the removal of affected animals (Sevik & Dogan, 2017). The treatment of LSD is only symptomatic and targeted at preventing secondary bacterial complications using a combination of antimicrobials, anti‐inflammatory, supportive therapy and anti‐septic solutions (Salib & Osman, 2011). The culling of affected animals, movement restrictions and compulsory and consistent vaccination have been recommended as control strategies (Beard, 2016; OIE WAHIS, 2016; Tuppurainen, Venter, et al., 2017). However, regarding the role of arthropod vectors, elimination of the disease is likely to be difficult and any delays in the removal of infected animals increase the risk of LSD transmission (Tuppurainen, Venter, et al., 2017). Moreover, risk factors should be considered in control activities (Sevik & Dogan, 2017). Educating veterinarians and livestock workers would enable them to perform timely diagnoses of clinical cases, helping to slow the spread of disease (Beard, 2016).

Members of the capripoxvirus are known to provide cross‐protection. Hence, homologous (Neethling LSDV strain) and heterologous (sheeppox or goatpox virus) live attenuated vaccines can all be used to protect cattle against LSD infection (OIE, 2013). In LSD‐free countries that use the sheeppox vaccine to protect sheep against sheep pox, it was recommended to use the same vaccine during LSD outbreaks because of potential safety issues associated with the live attenuated LSDV vaccine use (Tuppurainen & Oura, 2012). Furthermore, the rapid confirmation of a clinical diagnosis is essential so that eradication measures, such as quarantine, slaughter‐out of affected and in‐contact animals, proper disposal of carcasses, cleaning and disinfection of the premises, and insect control can be implemented as soon as possible during the eruption (Constable et al., 2017; Tuppurainen et al., 2005). Moreover, rigorous import restrictions on livestock, carcasses, hides and semen from endemic areas must be in place in disease‐free areas (Sevik & Dogan, 2017).

It is known that complete immunity against LSD was not provided by used sheep pox vaccines (Brenner et al., 2009). Nevertheless, they are used in some countries such as Iraq, Iran, Turkey and African countries with overlap between LSD, SPP and GTP (Sameea Yousefi et al., 2017).

The commercially accessible vaccines against LSD are live attenuated vaccines. Although cutaneous lesions have developed in some vaccinated animals after exposure to the virus, there were a greater amount of clinical cases in unvaccinated flock compared with vaccinated flock (Brenner et al., 2009; Stram et al., 2008). These cheap vaccines can give adequate protection through annual vaccination programmes (Tuppurainen, Venter, et al., 2017). Currently, the safety and efficacy of a newly developed inactivated vaccine have been confirmed in a field study by Hamdi et al. (2020).

Live vaccines produce a strong and long‐lasting immune response, and are efficient in the control of disease spread (Tuppurainen et al., 2020). However, live vaccines can cause local inflammation and a mild disease with skin lesions (Bedekovic et al., 2017). Although inactivated vaccines are costly and need several administrations, they are safe and it is possible to combine them with other antigens to make polyvalent vaccines that could be used in disease‐free countries. Moreover, inactivated vaccines could be applied in the final stage of disease eradication as a part of the strategy that uses live vaccines first (Hamdi et al., 2020).

As there is a chance of recombination between the wild field strain and the live vaccine, the risk of coinfection should be considered with the use of live vaccines (Sprygin et al., 2018). Natural infection is probably made worse by the vaccination of infected animals (Sprygin et al., 2019). Also, these vaccines are not recommended in disease‐free countries. A differentiating infected from vaccinated animals (DIVA) should be developed for non‐endemic countries, this would also be an effective tool for endemic countries (Tuppurainen, Venter, et al., 2017).

## 9 CONCLUSIONS

The recent spread of the disease into disease‐free areas indicates its epidemiological and economic significance. Considering the extensive boundaries of Middle East countries, animal movements among these countries should be attentively controlled by veterinary authorities. Furthermore, paying close attention to the different aspects of the disease, such as transmission and epidemiology, and the implementation of effective preventive measures such as vaccination, could result in better disease control. Therefore, accurate and timely diagnosis in endemic areas, vaccination with the homologous strain of the LSDV, vector control, animal movement restriction and LSDV testing of bulls used for breeding are highly recommended as tools to control further spread.

## CONFLICT OF INTEREST

The authors declare that there was no conflict of interest.

### Peer Review

The peer review history for this article is available at https://publons.com/publon/10.1002/vms3.434.
